# G_n_T Motifs Can Increase T:A→G:C Mutation Rates Over 1000-fold in Bacteria

**DOI:** 10.1093/molbev/msaf183

**Published:** 2025-08-04

**Authors:** James S Horton, Joshua L Cherry, Gretel Waugh, Tiffany B Taylor

**Affiliations:** Institut Cochin, Université Paris Cité, INSERM U1016, CNRS UMR 8104, Paris 75014, France; Department of Life Sciences and Milner Centre for Evolution, University of Bath, Claverton Down, Bath, UK; Division of Intramural Research, National Library of Medicine, National Institutes of Health, Bethesda, MD, USA; Division of International Epidemiology and Population Studies, Fogarty International Center, National Institutes of Health, Bethesda, MD, USA; Department of Life Sciences and Milner Centre for Evolution, University of Bath, Claverton Down, Bath, UK; Department of Life Sciences and Milner Centre for Evolution, University of Bath, Claverton Down, Bath, UK

**Keywords:** mutation hotspot, mutagenic nucleotide motif, local nucleotide context, homopolymeric tract, transversion mutation

## Abstract

Nucleotides across a genome do not mutate at equal frequencies. Instead, specific nucleotide positions can exhibit much higher mutation rates than the genomic average due to their immediate nucleotide neighbors. These “mutational hotspots” can play a prominent role in adaptive evolution, yet we lack knowledge of which short nucleotide sequences drive hotspots. In this work, we employ a combination of experimental evolution with *Pseudomonas fluorescens* and bioinformatic analysis of various *Salmonella* species to characterize a short nucleotide motif (≥8 bp) that can drive T:A→G:C mutation rates >1000-fold higher than the baseline T→G rate in bacteria. First, we experimentally confirm previous analysis showing that homopolymeric tracts (≥3) of G with a 3′ T frequently mutate so that the T is replaced with a G, resulting in an extension of the guanine tract, i.e. GGGT → GGGG. We then demonstrate that the potency of this T:A→G:C hotspot is dependent on the nucleotides immediately flanking the G_n_T sequence. We find that the dinucleotide immediately 5′ to a G_4_ tract and the dinucleotide immediately 3′ to the T strongly affect the T:A→G:C mutation rate, which ranges from ∼5-fold higher than the typical rate to over 1000-fold higher depending on the flanking elements. G_n_T motifs are therefore comprised of several modular nucleotide components which each exert a significant, quantifiable effect on the mutation rate. This work advances our ability to accurately identify the position and quantify the mutagenicity of hotspot motifs predicated on short nucleotide sequences.

## Introduction

Mutation is the ultimate source of genetic variation that facilitates evolution. However, as most mutations are deleterious, species have evolved various prevention and repair mechanisms to decrease the average genomic mutation rate to very low frequencies. Bacterial species have some of the lowest average genomic mutation rates, which are typically around 10^−9±1^ per nucleotide per generation (Lynch 2010). However, while the average rate may be very low, this is not true for all mutations. Instead, multiple biases skew the likelihood of observing particular mutations and cause some to happen much more frequently than others. For example, transition bias (Stoltzfus and Norris 2016) and A:T bias (Hershberg and Petrov 2010) skew which nucleotide substitutions are more likely to occur, and deletion bias (Kuo and Ochman 2009) skews DNA toward contraction rather than expansion. As well as biases in the types of mutations that are more common, certain genomic positions also exhibit higher mutation rates than others. For example, the center of the replichore has a higher mutation rate than the termini in multiple bacterial species (Dillon et al. 2018), as do regions near sites of double-stranded breaks (Shee et al. 2012). More locally, promoter regions for genes facing away from the replication fork can exhibit higher transition substitution and indel rates (Sabari Sankar et al. 2016). More locally still, a single-nucleotide can exhibit a mutation rate that is over a hundred times higher than a neighboring nucleotide (Schroeder et al. 2016). The primary determinant of this latter phenomenon is the local nucleotide sequence itself.

Local DNA sequence is one of the major drivers of mutation rate heterogeneity throughout genomes, which is the case in both bacterial and in human DNA (Schroeder et al. 2016; Liu and Samee 2023). Nucleotide motifs of just five nucleotides can generate local spikes in mutation rate, commonly referred to as “mutational hotspots”. These include recognition sequences for Dcm methyltransferase (i.e. CCWGG), which increase C:G→T:A transition rate at the second C position by ∼8-fold (Cherry 2018). A more potent hotspot motif is a homopolymeric tract or simple sequence repeat (e.g. CCCCC). These are hotspots for indel mutations that cause the tract to either expand or contract by one or more nucleotides. A homopolymer of length five increases indel rates by ∼100-fold, and the hotspot continues to increase in potency the longer the tract (Lee et al. 2012). The transition rate of a nucleotide can also vary by up to and over 100-fold depending on the single nucleotides situated either side of it, e.g. ACT versus GCG (Sung et al. 2015; Schroeder et al. 2016). Identifying the nucleotide sequences that generate mutational hotspots is therefore of central importance for predicting mutation rate heterogeneity throughout bacterial genomes. However, as not all nucleotide sequences that generate mutational hotspots are so readily identified, the challenge is to distinguish which nucleotide motifs impact local mutation rates.

Recent analysis of numerous species from the NCBI Pathogens database has found that T:A→G:C mutational hotspots in Pseudomonadota (Proteobacteria) and Bacillota (Firmicutes) are generated when a T residue is preceded by tracts of G, e.g. GGGT→GGGG (Cherry 2023). The potency of these hotspots increases with the length of the preceding guanine tract, with T:A→G:C rates on average being over 400-fold higher when a T is preceded by five or more G's relative to when it is preceded by two or fewer G's in *Salmonella*. This mutagenic effect is also much more pronounced when the G_n_T sequence is on the leading strand of DNA replication (Cherry 2023). Inspired by this finding, we first sought to confirm this result experimentally. Next, we wanted to ask whether G_n_T sequences could be used to accurately predict the mutagenicity of specific T:A→G:C mutational hotspots in bacterial genomes.

To accurately determine mutation rates at specific genomic positions, we needed to answer whether G_n_T sequences affect mutation in a vacuum or whether they are influenced by the local nucleotide context. We answered this by complementing genome-wide analysis of *Salmonella* with an experimental approach using *Pseudomonas fluorescens*, where we examined the mutation rates at specific G_n_T sequences with different local nucleotide contexts. Using this two-pronged approach, we characterize a novel nucleotide motif that increases T:A→G:C mutation rates in bacteria by up to and over a factor of 1000 when on the leading replicative DNA strand. We show that T:A→G:C substitution mutational hotspots are generated by a ≥ 8 bp motif. The central component of this motif is the G_n_T sequence, and we observe that G tract length is strongly positively correlated with increased T:A→G:C mutation rates. However, we also find that whether the G_n_T sequence operates as a mutational hotspot is determined by local nucleotide context, specifically the immediate flanking nucleotides. Rates of mutation are influenced by the 3′ dinucleotide following the T and by the 5′ dinucleotide immediately preceding the G tract, making these key components of what we hereafter label the G_n_T motif. We combine our experimental and analytical data to show that the impact on T:A→G:C mutation rates with different combinations of flanking nucleotides is similar in our two species. This suggests that the mutation rates for G_n_T motifs determined for this study will be good predictors of hotspot strength in other species of bacteria, particularly other Gammaproteobacteria.

## Results

### Homopolymeric Tracts of Guanine With a 3′ Thymine are Primary Components of T:A→G:C Mutational Hotspots

We employed an immotile variant of the soil bacterium *P. fluorescens* SBW25 to investigate G_n_T mutational hotspots experimentally. When grown on soft agar, populations of this strain are able to rapidly re-evolve the motility phenotype (Taylor et al. 2015). We previously observed that most independent replicates re-evolve motility by fixing the same de novo mutation, found at a mutational hotspot within the gene *ntrB*. This hotspot, A:T→C:G at position 289, is sensitive to synonymous nucleotide variation, as the introduction of six synonymous changes within 15 bp of position 289 removed the hotspot in SBW25 (Horton et al. 2021). The local nucleotide context neighboring the A:T hotspot position was therefore demonstrated to be integral to hotspot potency, but the components of the nucleotide motif that drove the hotspot remained unknown.

The gene *ntrB* is natively encoded on the lagging strand, but from the perspective of the leading strand during replication the mutational hotspot nucleotide sequence 5′-3′ is: GGGGT, which mutates to become GGGGG. Therefore, this experimentally determined T:A→G:C mutational hotspot may have been an example of the G_n_T phenomenon observed within the NCBI Pathogens dataset. The first objective of the current study was to determine if guanine tract length was the primary facilitator of the mutational hotspot within *ntrB*. By doing so, we would provide experimental evidence of the widespread mutational hotspot phenomenon observed throughout many bacterial taxa (Cherry 2023).

**Fig. 1.**
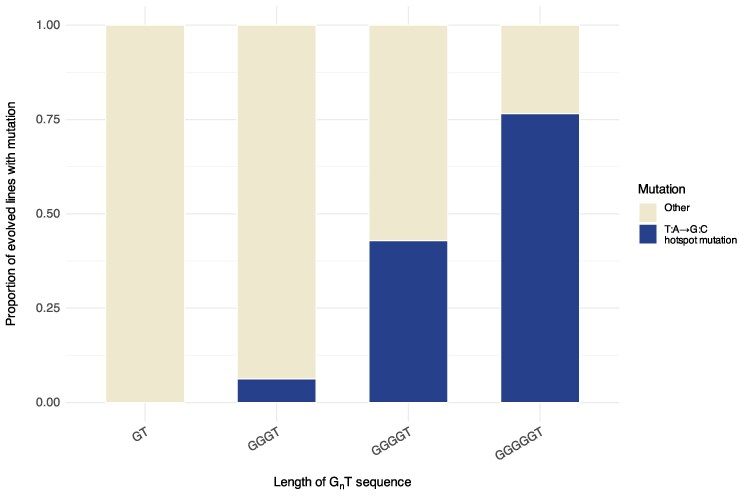
T:A→G:C mutation frequency is positively correlated with length of the preceding G tract. Ten *ntrB* variants with synonymous nucleotide changes neighboring a T position were used to study the T:A→G:C hotspot motif. Each motif variant contained a homopolymeric tract of guanine 5′ to the T that was either 1, 3, 4, or 5 bp in length. Shown are the aggregated proportion of evolved mutant lines that acquire a T:A→G:C hotspot mutation at the thymine position when it is preceded by 1 G (*n* = 29), 3 G's (*n* = 112), 4 G's (*n* = 434), or 5 G's (*n* = 47). Total sequenced replicates = 622. Overall, preceding G tract length is a strong predictor of T:A→G:C mutational hotspot potency (linear regression model, *R*^2^ = 0.82).

Our approach for measuring changes in mutational hotspot potency was simple: we re-evolved motility in immotile derivatives of *P. fluorescens* SBW25 and counted how often the hotspot mutation was observed. The motility phenotype can be acquired through a single de novo mutation from a pool of possible mutations in multiple loci, including the T:A→G:C mutation of interest. We assessed hotspot potency by measuring the proportion of independent replicates that acquired the T:A→G:C mutation as opposed to other viable mutations within the same gene or elsewhere. We then made synonymous changes to the nucleotide motif immediately surrounding the T:A position of interest, such as the neighboring G tract, and repeated the experiment. If the proportion of independent replicates that acquired the T:A→G:C hotspot mutation increased, this showed an increase in mutation rate at the motif; if it decreased, then the reverse was true (Fig. 1).

For all but one strain, the only difference between the hotspot variants evolved for this study was 1 to 9 synonymous nucleotide variations within the gene *ntrB* that changed the local nucleotide sequence neighboring the thymine position(s) of interest. Among the introduced synonymous changes were those that changed the length of the preceding G tract (the other introduced variations are explained in more detail in the next section). We first pooled the results for all variant motifs surrounding a mutable T, then looked at their hotspot potencies with respect to the number of G's preceding the thymine. We observed a positive correlation between T:A→G:C hotspot mutation rate and the length of the preceding G tract (Fig. 1; *R*^2^ = 0.82). Our dataset therefore experimentally corroborates the previous analysis in *Salmonella* and additionally supports that the *ntrB* T:A→G:C hotspot is a product of a G_n_T motif.

### T:A→G:C Hotspot Motifs in Experimental Populations of *P. fluorescens* are Contingent on Preceding G Tract Length, the Flanking 5′ Dinucleotide, and Flanking 3′ Nucleotide

**Fig. 2.**
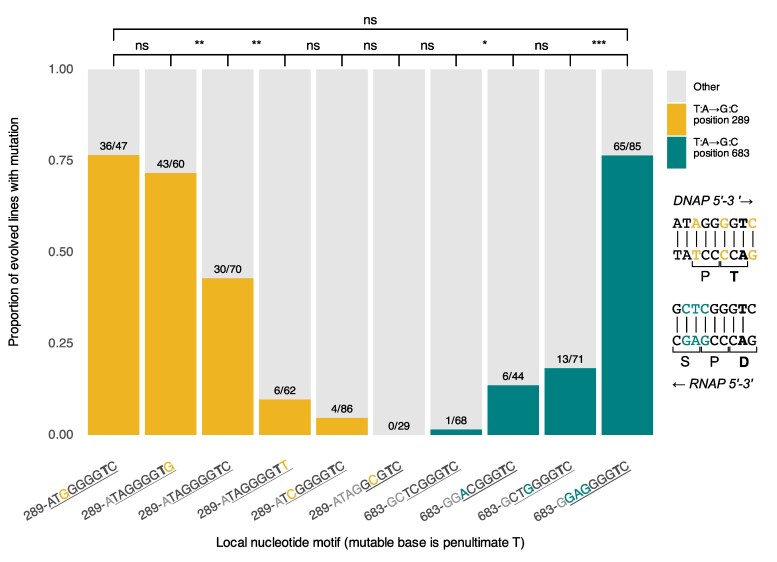
The frequency of T:A→G:C mutation in *P. fluorescens* is strongly influenced by neighboring G tract length and flanking nucleotide sequence. T:A→G:C hotspot motifs were augmented around two thymine positions in the gene *ntrB*: coding position 289 (yellow bars, left) and coding position 683 (teal bars, right). Nine nucleotides neighboring positions 289 and 683 are shown on the *x*-axis, listed 5′-3′ with respect to leading strand replication. The nucleotides observed to influence mutation rate in this study are underlined, these are: the dinucleotide 5′ to the guanine tract, a homopolymeric tract of guanine (1 to 5 bp in length), the hotspot T position that mutates into G (bold), and the nucleotide 3′ to the mutable thymine. Nucleotides outside the investigated G_n_T motif are grayed. Positions that were synonymously changed for each variant (relative to the wild type sequences: bars 3 and 7, right panel) are highlighted by color. The right panel highlights all sites that were targets of synonymous mutation and shows the direction of replication (DNAP) and transcription (RNAP). Nucleotide 289 encodes a first codon position and 683 a second codon position, and the two neighborhoods encode different amino acids (right panel). This facilitates unique motif changes around the two sites. The *y*-axis shows the proportion of independent replicates that acquired motility via the T:A→G:C hotspot mutation, calculated from the observed counts plotted over each bar. Significance values were determined using Fisher's exact test (* *P* < 0.05; ** *P* < 0.01; *** *P* < 0.001).

Having shown the importance of preceding homopolymeric tracts of guanine for T:A→G:C mutational hotspots, we next asked whether the motif was affected by other nucleotides in the immediate neighborhood. We assessed this question in two ways. First, the coding sequence around the *ntrB* 289 hotspot has considerable codon flexibility, which allowed us to probe the impact of the immediate flanking nucleotides. We constructed strains with synonymous substitutions at the second position from T in the G tract (natively G_4_T); the nucleotide 5′ to the G tract; and the nucleotide 3′ to the mutable T, i.e. NGGNGTN (*N* = any nucleotide). Second, we wanted to confirm that any determined hotspots exhibited high mutation rates only because of the short sequence of nucleotides we augmented and were not contingent on the wider nucleotide neighborhood. To do this, we selected an alternative coding A:T position—683 in *ntrB*—which also facilitates motility following an A:T→C:G mutation (Horton et al. 2021). This position natively has three preceding G's, which we were able to extend synonymously. Additionally, we were able to synonymously mutate the 5′ dinucleotide, i.e. SWNGGGTC (S = G/C, W = A/T). The native nucleotide sequences neighboring positions 289 and 683 are displayed visually on Fig. 2. In total, we engineered and evolved ten hotspot motif variants across the two hotspots positions to determine each nucleotide component's impact on hotspot potency (Fig. 2).

Our results reveal that the modular components flanking the core of the hotspot motif—the 5′ dinucleotide and the 3′ nucleotide—exert a significant effect on the mutational hotspot. We evolved three variants of the 289 hotspot that had identical 5′ TA dinucleotides and G tract lengths of four (TAG_4_T), with the only difference being the 3′ nucleotide following the mutable T (Fig. 2, bars 2 to 4). We observed that hotspot potency significantly decreased as the 3′ nucleotide changed from G to C to T (Fisher's exact test, *P* values < 0.0014). Similarly, two motifs around the 289 hotspot that only varied at the 5′ nucleotide position (TNG_4_TC; Fig. 2, bars 3 and 5) revealed that a 5′ A is significantly more mutable than a 5′ C (Fisher's exact test, *P* < 0.001). Finally, two CG_3_TC motifs at the 683 hotspot significantly differed in hotspot potency when the second 5′ nucleotide (the −2 position, i.e. WCG_3_TC) changed from T to A (Fig. 2, bars 7 to 8; Fisher's exact test, *P* < 0.015). The −2 nucleotide was also found to exert a significant effect on AG_4_TC at the 289 and 683 hotspots, as G was significantly more mutable than T (Fig. 2, bars 3 and 10; *P* < 0.001).

These results show that the immediate flanking nucleotides are significant components of G_n_T motifs. Additionally, our building of a hotspot at position 683 demonstrates that potent hotspot motifs can be generated in different wider nucleotide contexts. Outside of the 8 bp motif window, the regions around positions 289 and 683 share very little sequence similarity (±6 bp contains 2/12 base identity). Yet we were able to increase mutation rate at position 683 to surpass the native 289 position by augmenting only the established G_n_T motif nucleotides. We observed that hotspot potency was significantly increased at position 683 by either changing the −2 nucleotide or extending the G tract length to four (Fig. 2, bars 7 to 9; Fisher's exact test, *P* < 0.015). The G_4_ variant, however, remained significantly less mutable than the native 289 hotspot (Fig. 2, bars 3 and 9; Fisher's exact test, *P* < 0.01) until we also changed the 5′ dinucleotide from CT to GA. After this change the 683 hotspot exceeded the native 289 hotspot sequence (Fig. 2, bars 3 and 10; Fisher's exact test, *P* < 0.001). Therefore, by only augmenting the short nucleotide motif of 7 to 9 bp (depending on G tract length), we are able to build T:A→G:C hotspots within multiple nucleotide neighborhoods.

### T:A→G:C Hotspot Motif Analysis in Natural Populations of Salmonella Shows that Flanking Nucleotides Determine Whether Mutation Rates are ∼5-fold or >1000-fold Above the Genomic Baseline

**Fig. 3.**
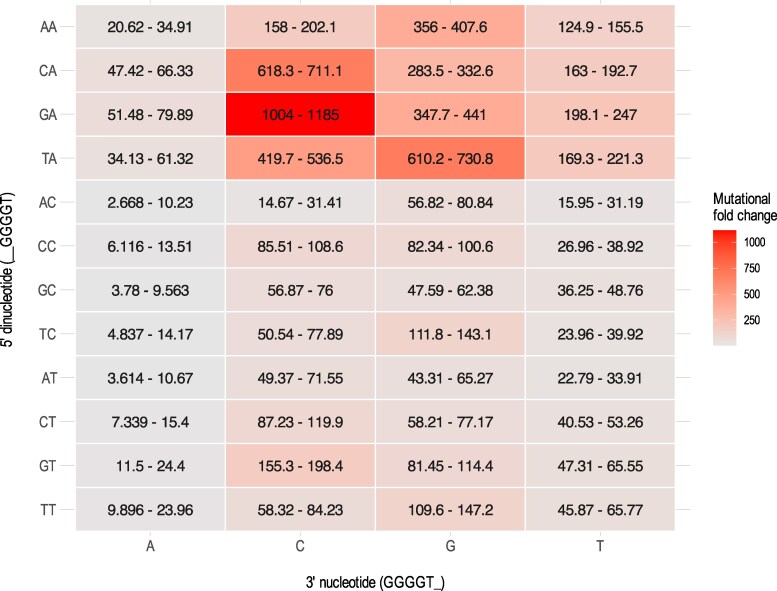
The impact of flanking nucleotides neighboring G_4_T sequences on T:A→G:C mutation rates in natural *Salmonella* populations. A heatmap showing the T:A→G:C mutation rate for G_4_T motif variants relative to the average T:A→G:C genomic mutation rate with no preceding Gs. Each panel represents a unique combination of nucleotides (5′ dinucleotide on the *y*-axis and 3′ nucleotide on the *x*-axis) flanking a G_4_T sequence. Panels are colored according to mean mutational fold change and annotated with 95% confidence intervals.

**Fig. 4:**
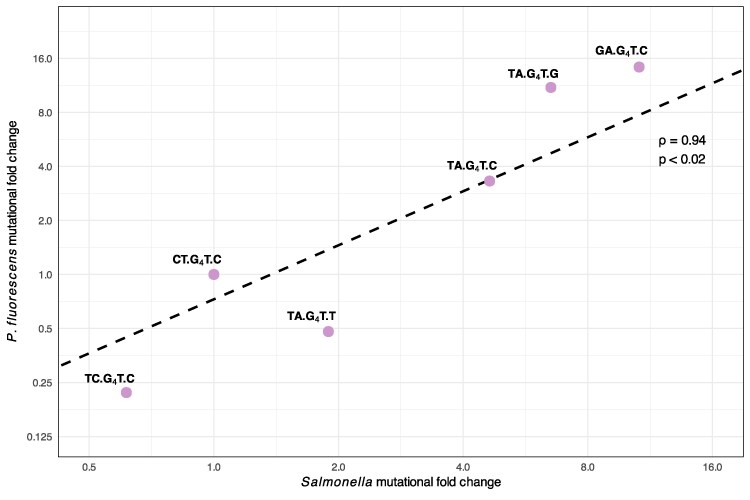
Relative mutation rates of G_4_T motifs in *P. fluorescens* are highly predictive of those in *Salmonella*. An experimental P*. fluorescens* dataset and analysis of natural *Salmonella* populations were made comparable by determining mutational fold changes of G_4_T motifs (labels) relative to the motif CTG_4_TC (CT.G_4_T.C). Points show mutational fold changes of *P. fluorescens* motifs (*y*-axis; determined by Fisher's exact test odds ratio) and fold changes observed for each motif in *Salmonella* (*x*-axis; see Materials and Methods). The line represents the least squares fit in the log domain. Values for rho and *P* were determined by a Spearman rank-order correlation.

Our experimental assay with *Pseudomonas fluorescens* was able to demonstrate whether a nucleotide substitution within a T:A→G:C hotspot motif exerted a significant effect on hotspot potency. This assay is however, limited, primarily by sample size, in its ability to reveal differences between hotspots with low mutation rates and sometimes between those with similar mutation rates. Sample size also permitted only rough estimates of relative mutation rates among motifs. Fortunately, we were able to reinforce our experimental data by re-analysing the *Salmonella* dataset that was generated by (Cherry 2023) in order to accurately estimate the mutation rate for each unique G_n_T motif. This dataset includes nearly 1.5 million single-nucleotide changes (over 100,000 T→G changes) inferred from genome sequences of hundreds of thousands of *Salmonella* isolates. We adapted the original script from (Cherry 2023) to estimate mutation rates for extended motifs that included the flanking nucleotides we examined experimentally. As most of our experimental data focussed on G_4_T motifs we focus on this tract length for our analysis (Figs. 3 to 4), but we also determined T:A→G:C mutation rates for G_3_T and G_5_T motifs (supplementary figs. S1 to S2, Supplementary Material online).

Our analyses found that mutation rates differed by orders of magnitude depending on the flanking nucleotides. At the lowest end, flanking nucleotides of 5′ AC and 3′ A almost entirely eliminate the G_4_T motif hotspot, with T:A→G:C rates being only ∼5.6-fold higher than the baseline rate, i.e. the genomic T:A→G:C rate when there are no preceding G's (95% CI = 2.668 to 10.23; Fig. 3). At the high end, flanking nucleotides of 5′ GA and 3′ C lead to T:A→G:C mutation rates ∼1112-fold higher than the genomic baseline (95% CI = 1004 to 1185; Fig. 3). In general, the mutation rate is highest when the 3′ nucleotide is a C, then G, then T, then A. For the 5′ nucleotide, the reverse is true, with the highest mutation rates being associated with A, then T, then C.

The influence of the −2 nucleotide is more variable and demonstrates the context-dependency of motif mutagenicity. For example, 5′ dinucleotide GA typically increases mutation rates most, but TA is the most mutable when the 3′ nucleotide is a G (Fig. 3). This finding is corroborated by our experimental data, as the mutation rate of 5′ TA, 3′ C is significantly lower than both 5′ TA, 3′ G and 5′ GA, 3′ C (Fig. 2, bars 2 to 3 and 10). We additionally observed that the relative impact of the flanking nucleotides on hotspot potency varies depending on G tract length. For motifs with 3′ C, the most mutable 5′ dinucleotide is AA for G_3_ (supplementary fig. S1, Supplementary Material online), GA for G_4_ (Fig. 3), and CA or TA for G_5_ (supplementary fig. S2, Supplementary Material online). Similarly, for motifs with 5′ TA, the most mutable 3′ nucleotides are C then G for G_3_ (supplementary fig. S1, Supplementary Material online), G then C for G_4_ (Fig. .3), and C then T for G_5_ (supplementary fig. S2, Supplementary Material online).

We next scrutinized the relationship between our datasets by comparing laboratory estimates of relative mutation rates for various motifs in *P. fluorescens* to the estimates obtained from the analysis of natural *Salmonella* isolates (Fig. 4). The relative rates correlate well between the two organisms (Spearman's rank order correlation, rho = 0.94, *P* < 0.02). This correlation coefficient is expected to be an underestimate due to uncertainty in the rate estimates, especially the large uncertainties observed for our *P. fluorescens* data. Despite this, the two datasets exhibited highly similar mutation rate estimates for the set of motifs we analysed.

One final restriction of our experimental assay is that we had limited opportunity to explore the second 3′ nucleotide (the +2 position) through synonymous manipulation. We were however, able to analyse the impact of this nucleotide position by further partitioning our *Salmonella* dataset (supplementary fig. S3, Supplementary Material online). We split our data to consider two flanking nucleotides upstream and downstream of G_4_T sequences. At this level of partitioning our confidence intervals widen, and not all flanking nucleotide combinations are represented. Regardless, the +2 nucleotide is also predicted to have a significant impact on mutation rate. In the most notable example, 5′ GA and 3′ C (GAG_4_TC, the most mutagenic G_4_ flanking nucleotide combination examined experimentally) is predicted to change mutation rates by ∼360-fold if +2 nucleotide is an A, but ∼2700-fold if the nucleotide is a G (supplementary fig. S3, Supplementary Material online). The predicted pattern of mutagenicity is an echo of the 3′ nucleotide position, in that mutation rates are highest when the +2 nucleotide is a C or G, then T, then A. While we did not verify these findings experimentally, the +2 nucleotide is likely an element of the G_n_T motif. Full mutation rate predictions for 5′ and 3′ dinucleotides flanking G_4_ motifs are provided by supplementary fig. S3, Supplementary Material online.

Overall, the *Salmonella* analyses reinforce that the flanking nucleotides are important determinants of mutation rates at G_n_T motifs, and the data provide accurate quantifications of how the flanking nucleotides impact mutation rates. The analyses also show that the effects of the 5′ dinucleotide, G tract length, and 3′ dinucleotide on T:A→G:C mutation rate are not independent. Rather, the effect on mutation rate of changing one component of the motif is contingent on the other components.

### Multiple G_n_T Motifs Facilitate More Rapid and More Predictable Evolutionary Outcomes

Using our experimental system, we had successfully generated two potent G_n_T hotspot motifs through synonymous mutation of *ntrB*. Individually, these motifs drive evolution to become more predictable, as mutations are observed more often at the hotspot site in *ntrB* rather than within other genes, such as *glnK* or *glnA* (Horton et al. 2021; Shepherd et al. 2022). We asked if implementing two G_n_T motifs within the same reading frame would further increase evolutionary predictability at the level of the locus. Additionally, as the hotspot motifs increase the rate at which mutations are acquired, we sought to demonstrate that multiple G_n_T motifs can also increase the speed of adaptive evolution.

We investigated this by evolving a strain with two hotspot motifs in the *ntrB* gene, both a modified hotspot around nucleotide 289 and an engineered hotspot around position 683. We chose the two most potent G_n_T motifs generated at each position: 289-ATG_5_TC, and 683-GAG_4_TC. These motifs exhibit similar, but nonidentical, mutation rates that are approximately within 2.5-fold of one another (*P. fluorescens* data (Fig. 2), Fisher's exact test = 1:1, 95% CI = 0.4 to 2.6).

**Fig. 5.**
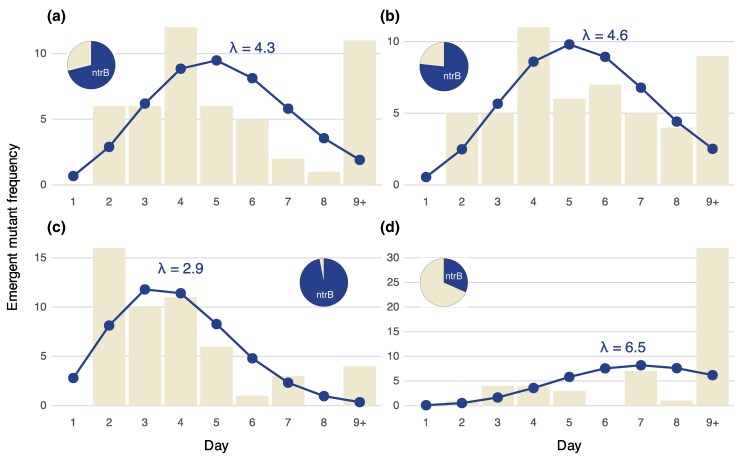
The number and orientation of T:A→G:C hotspot motifs determine the rate of evolution and evolutionary predictability. Emergence of the motility phenotype and the proportion of evolved replicates with de novo mutation in *ntrB* were determined for four hotspot motif *ntrB* variants: a) 289-ATG_5_TC encoded on the leading strand; b) 683-GAG_4_TC encoded on the leading strand; c) 289-ATG_5_TC and 683-GAG_4_TC encoded on the leading strand; d) 289-ATG_5_TC and 683-GAG_4_TC encoded on the lagging strand. Each variant was used to seed a batch of independent replicates (*n* = 49, 52, 52, and 51 respectively). These were placed under selection for motility and monitored for 8 days (*x*-axis). The frequency of the emergent motility phenotype was recorded (*y*-axis) and replicates that did not evolve within the experimental window were designated to have evolved in 9+ days. Mean emergence times were determined by plotting a Poisson distribution. Replicates that evolved within 8 days were sent for Sanger sequencing of the *ntrB* locus (*n* = 38, 43, 44, and 19). The proportion of replicates that acquired a de novo mutation in *ntrB* are shown by the pie charts within each panel.

For this experiment, we recorded the proportion of motile replicates with mutation in *ntrB* as well as the time taken to evolve the motility phenotype (Fig. 5). The replicate populations containing a single hotspot evolved similarly, taking an average of ∼4.5 d to evolve and doing so via *ntrB* mutation in ∼75% of instances (Fig. 5a, b). As expected, we observed that evolution happened significantly faster and became more predictable at the level of locus when populations had both hotspots (Fig. 5c). Populations evolved in <3 d on average, and mutations were observed in *ntrB* in ∼95% of instances. As such, the speed of adaptation and the mutability of a locus can be compounded by modifying a G_n_T motif to be more potent and by adding further motifs into the reading frame.

Finally, we also flipped the directionality of the double hotspot *ntrB* gene to show that the mutability of G_n_T motifs is highly sensitive to their orientation with respect to leading strand replication. When *ntrB* was encoded on the opposite strand the average time to evolve motility was 6.5 d, which is likely an underestimate as most replicates did not evolve during the eight-day experimental window (Fig. 5d). Of those that did evolve, mutations in *ntrB* were observed in ∼20% of instances. This result confirms previous observations that for potent hotspot activity, a G_n_T motif must be encoded 5′-3′ on the leading strand of replication (Shepherd et al. 2022; Cherry 2023).

## Discussion

Mutation rates across a genome are shaped by multifaceted mechanisms, resulting in significant variation rather than uniformity. Specific sites, known as “mutational hotspots,” exhibit mutation rates much higher than the genomic average and can influence both the direction and predictability of bacterial adaptation (Horton and Taylor 2023). While it's known that local nucleotide sequences impact mutation rates (Sung et al. 2015; Schroeder et al. 2016), the specific nucleotide combinations that generate hotspots are not well understood. In this study, we employed experimental and bioinformatic methods to characterize a short nucleotide motif (G_n_T) that can increase T:A→G:C mutation rates by over 1000-fold in bacteria. Through experimental evolution with *P. fluorescens* and complementary bioinformatic analysis of *Salmonella* species, we build on previous work that found that T:A→G:C mutation hotspots are generated by homopolymer G tracts with a 3′ T (Cherry 2023) to show that G_n_T motifs depend on homopolymeric G tracts in combination with the flanking nucleotides. These findings highlight the modularity of hotspot components, providing insights that can enhance predictive models of mutation rate variability.

### G_n_T Motifs as Biasing Agents in Experimental Evolution

We found that the mutational hotspot in *ntrB* is a product of a G_n_T motif, which raises the question as to whether G_n_T hotspots in other genes have also influenced adaptive evolution. Multiple independent studies using *P. fluorescens* and *P. protegens* strongly suggest that this is the case. T:A→G:C mutational hotspots have been found in the genes *awsR*, *fuzY*, and *wspF*, and these can all be explained by G_n_T motifs situated around each hotspot site (Ferguson et al. 2013; Lind et al. 2019; Pentz and Lind 2021). For example, mutations in *fuzY* facilitate the fuzzy spreader phenotype in *P. fluorescens* SBW25. Ferguson et al. reported that any of 59 unique mutations within this gene can cause the phenotype, 23 of which are unique base substitutions. Yet 25% of all replicates (28/112) acquired an identical T:A→G:C transversion at the same position (T443G), which could not be explained by selection. This bias is instead likely explained by the G_n_T motif around position 443 (TCG_4_TG), which is estimated to mutate ∼129-fold more often than the genomic baseline. Additionally, the frequently observed mutation *awsR* A79C (Lind et al. 2019) can be explained by the leading strand motif CAG_3_TC around position 79, and the frequently observed mutation *wspF* T812G (Pentz and Lind 2021) can be explained by the motif GTG_5_TG around position 812. We have also previously observed the mutation *rpoN* (σ^54^) A688C (Horton et al. 2021), which similarly can be explained by the G_n_T motif CAG_5_TC around position 688. Together, these four studies support that G_n_T motifs frequently influence adaptive evolutionary trajectories during experimental evolution.

### Predicting and Controlling Mutagenesis

A key advantage to identifying the context-dependency of hotspot motifs and quantifying their impact on hotspot potency is that it allows us to accurately predict mutation rates. It is reassuring that the relative mutation rates of G_n_T motifs determined from *Salmonella* are applicable to *P. fluorescens*, a distantly related species in the Gammaproteobacteria class (Williams et al. 2010). This suggests that predicting mutation rates at G_n_T hotspot motifs using the *Salmonella* data will be reasonably accurate at identifying the location of potent T:A→G:C hotspots across broad genomic backgrounds. Additionally, due to their short sequence, mutable G_n_T motifs are common features in the coding regions of bacterial genomes. Our data do not support that G_n_T mutation rates are correlated with genomic GC-content, but these motifs do appear more often in GC-rich genomes such as that of *P. fluorescens* (supplementary fig. S4, Supplementary Material online). Above, we shared examples of G_n_T motifs within multiple genes in *P. fluorescens* and *P. protegens*. These multiple independent observations are perhaps not surprising when we consider that there are 1924 naturally occurring G_3-5_T motifs on the leading replicative strand of *P. fluorescens* SBW25 with forecast mutation rates >100-fold above the genomic baseline. And of these, ≥1238 genes (∼20% of reading frames) possess at least one hotspot motif that would result in a nonsynonymous T:A→G:C substitution (supplementary fig. S4, Supplementary Material online; see Materials and Methods). Assuming mutation rates are otherwise equal, we can predict that these reading frames will exhibit higher rates of nonsynonymous mutation than other genes and therefore may disproportionally contribute to adaptive evolutionary events.

As well as occurring naturally in many coding sequences, G_n_T motifs are so short that they can also be readily engineered into circuits to help control the evolvability of a system (e.g. Figure 5). Similar approaches could be used to introduce multiple destabilizing mutation hotspots in a coding sequence of interest, increasing the rate at which the locus will acquire a loss of function mutation. Or a hotspot could be used for gain of function mutation by increasing the removal rate of a premature stop codon (e.g. TGA→GGA). In summary, there is a growing interest in our ability to both forecast and exert control over the evolutionary process (Lind et al. 2019; Castle et al. 2021). G_n_T motifs, as agents that heavily bias the process of mutation, have the potential to contribute to both goals.

### Additional Molecular Factors Influencing G_n_T Motif Mutation Rates

The potencies of hotspots driven by short nucleotide sequences are often influenced by other genomic features, such as genomic position and DNA structure (Shepherd et al. 2022; Liu and Samee 2023). Recent work has also shown that DNA replication error-hotspots are affected by multiple genomic properties that fluctuate throughout the genome (Hasenauer et al. 2024). Therefore, identical G_n_T motifs within the same genome may not mutate at identical rates. The previous *Salmonella* analysis examined rates of T:A→G:C accumulation at G_n_T motifs as a function of chromosome position and found a roughly uniform distribution (Cherry 2023). This suggests that there is no strong systematic dependence of hotspot potency on genome position. However, our previous work found that the *ntrB* G_n_T motif is more potent when situated in its native position as opposed to the Tn7 insertion site, which is situated on the other replichore and is closer to the replication origin (Shepherd et al. 2022). Therefore, there may be instances where hotspot potency changes by perhaps an order of magnitude (using the data from Shepherd et al. 2022, as a guide) depending on genomic context. However, this phenomenon does not appear to be a simple effect of distance from the origin of replication.

### Possible Mechanism

The mechanism driving high mutation rates at G_n_T motifs is unclear. However, the central role played by the homopolymeric tract (also referred to as a simple sequence repeat) suggests slipped-strand mispairing as a causal factor. During DNA replication, dissociated parent and nascent DNA strands can re-anneal out of register due to homology with other nucleotides in the repeat region (Levinson and Gutman 1987). This process typically results in an indel mutation as the out-of-register mispairing causes a nucleotide to loop out of either the nascent or template strand, causing an insertion or deletion of the repetitive nucleotide respectively (Fan and Chu 2007). In this study we observe a substitution mutation, but this may be a product of slipped-strand mispairing if the T→G mutation is caused by insertion of a G followed by another dissociation event and re-pairing in the original register, resulting in a G:A mispair. If extension then continues and the mismatch is not repaired, the result will be a substitution of the original T:A hotspot position with its replacement G:C.

It is not clear how the flanking nucleotides contribute to the proposed slipped-strand events, although there are several possibilities. Short nucleotide sequences that generate mutational hotspots during DNA replication must overcome multiple barriers. A sequence must initially exploit error-prone features of the replicating polymerase to generate a mismatch or indel, and subsequently this error must evade proofreading and mismatch repair to ensure that the mutation becomes immortalized. The flanking nucleotides may contribute to mutagenicity during this process, either by facilitating the slipped-strand mispairing events—by making the DNA sequence more prone to opening or “breathing” (Oron-Karni et al. 1997; Jose et al. 2009)—or by aiding the resultant error to remain unrecognized by DNA proofreading and repair enzymes.

Proofreading by DNA polymerase discriminates mismatches by checking for correct Watson-Crick geometry (Bębenek and Ziuzia-Graczyk 2018), likewise mismatch repair recognizes errors based on DNA secondary structure (Pavlova et al. 2020). Previous works have found direct and indirect evidence that immediate flanking nucleotides can help stabilize mismatches (Allawi and SantaLucia 1998; Long et al. 2018), and that the structure of the DNA helix can be impacted several nucleotides away from a mismatch (Rossetti et al. 2015). We estimated melting temperatures for all 48 variations of 5′ dinucleotide, 3′ nucleotide G_4_T motif sequences assuming a G:A mismatch. When we controlled for the 5′ nucleotide we found a significant positive correlation between DNA stability and mutation rate for the second 5′ nucleotide and 3′ nucleotide (supplementary fig. S5, Supplementary Material online). Conversely, when we controlled for the 3′ nucleotide we observed a mostly nonsignificant but negative correlation between stability and mutation rate for the 5′ nucleotide and second 5′ nucleotide (supplementary fig. S5, Supplementary Material online). This relationship can be explained by the preference for C:G/G:C at the 3′ position and against C:G/G:C at the 5′ position. Therefore, the flanking nucleotides may impact stability in different ways, e.g. the low stability of the 5′ dinucleotide may facilitate DNA dissociation and breathing, while the 3′ nucleotide may stabilize the DNA double helix following a return to an in-register mismatch, helping the error to evade proofreading and repair.

If DNA stability is an important factor in hotspot potency, we can speculate further and note that the purine-purine G:A pair exhibits unique undertwisting of the DNA helix relative to other mismatches (Rossetti et al. 2015), which may help to explain why the homopolymer substitution phenomenon appears to be unique to G_n_T motifs (Cherry 2023). It has also been suggested that dNTP availability for replicating the “next nucleotide”, i.e. the 3′ nucleotide in this case, can influence the chance of proofreading. dNTPs in excess are thought to minimize proofreading opportunity and consequently increase mutation rate (Kumar et al. 2010; Schaaper and Mathews 2013). This could be playing a role in this system if dCTP/dGTP are consistently in excess. Finally, while documentations of sequential indel and mismatch events resulting from strand-slippage are rare, at least one study has previously reported on the phenomenon and implicated DNA breathing in facilitating the effect (Oron-Karni et al. 1997).

## Materials and Methods

### Strains and Culture Conditions

Experiments were performed using populations derived from *Pseudomonas fluorescens* SBW25 strain AR2 (SBW25ΔfleQ IS-ΩKm-hah: PFLU2552), which lacks functionality of FleQ and ViscB, rendering populations immotile (Alsohim et al. 2014). The strains constructed from this genomic background contain synonymous nucleotide substitutions in the gene *ntrB*, which has previously been found to resurrect motility under directional selection following a one-step de novo mutation in the coding sequence. *P. fluorescens* populations were cryogenically stored at −70 °C and grown at 27 °C; overnight cultures were agitated at 180 rpm. *E. coli* strains used for cloning (DH5α), conjugal transfer (SP50 pRK2073), transposon-mediated genome incorporation (S17 pTNS2), and allelic exchange (ST18) were cryogenically stored at −70 °C and grown at 37 °C; overnight cultures were agitated at 200 rpm.

Antibiotics used in this study were prepared at the following working concentrations: 50 μg/mL kanamycin sulfate; 100 μg/mL ampicillin sodium salt; 30 μg/mL gentamicin sulfate; 10 μg/mL tetracycline hydrochloride; 250 μg/mL streptomycin sulfate salt. All antibiotics were purchased from Sigma-Aldrich.

### Synonymous Manipulations of ntrB

To generate synonymous motif variants, we amplified the *ntrBC* operon from *P. fluorescens* SBW25 derivatives (NCBI Assembly: ASM922v1, GenBank sequence: AM181176.4) using outside primers ntrBC-HindIII-F and ntrBC-SacI-R (supplementary table S1, Supplementary Material online). In the first PCR, we generated two fragments using the outside primers in combination with complementary oligonucleotides that had approximately 40 bp homology overlapping the nucleotide sequence around *ntrB* position 289 or 683, depending on the desired construct (supplementary table S1, Supplementary Material online). In the second PCR, we annealed these two fragments with complementary overhangs to generate a complete *ntrBC* operon with synonymous variations at positions neighboring 289 or 683. For *ntrB* 289 variants we used *P. fluorescens* SBW25 strain AR2 as template DNA; for *ntrB* 683 variants, we used *P. fluorescens* SBW25 strain AR2-sm (Horton et al. 2021) as template DNA to remove the native hotspot at *ntrB* 289. All changes made to the nucleotide sequence around the T positions of interest left the encoded protein sequence unchanged. Most were single-base changes at third positions of codons. The exception was a change of AGC, which encodes serine, to TCC, which also encodes serine.

### Genomic Integration of Variant Sequences Through Mini-Tn7 Mediated Transposition

All *ntrBC* operon variants, aside from 289-TAGGCGTC, were integrated into a nonmotile *P. fluorescens* SBW25 derivative AR2 Δ*ntrBC* genomic background using mini-Tn7 mediated transposition (Choi and Schweizer 2006). As the native operon has been deleted in the host genomic background this incorporation operates as a functional translocation. Generated *ntrBC* inserts and the plasmid pJM220 (Meisner and Goldberg 2016) were digested with the restriction enzymes HindIII-HF and SacI-HF (NEB) for 1 h at 37 °C. This was followed by a 30-min digestion at 37 °C where RSAP (NEB) was added to the pJM220 digestion mix. The vector and insert were gel-extracted and purified with a Monarch DNA gel extraction kit (NEB) and the fragments were annealed using T4 DNA ligase (NEB) ligation at 16 °C for 12 h, followed by 4 °C hold for ∼4 h. The ligated plasmid was transformed into cloning *E. coli* strain DH5α and plated onto LB agar containing ampicillin and gentamicin. Conjugal transfer was performed by preparing 10 mL overnight LB broth cultures of the recipient *P. fluorescens* strain (supplemented with kanamycin), DH5α pJM220-*ntrBC* (supplemented with ampicillin and gentamicin), and the helper plasmids SP50 pRK2073 (supplemented with streptomycin) and S17 pTNS2 (supplemented with ampicillin). 700 μL of the *P. fluorescens* culture was combined with 100 μL of each *E. coli* strain to produce 1 mL final volume. The mixed population was pelleted through centrifugation at 8000 rcf for 3 min, the supernatant removed, and re-suspended in 1 mL fresh LB broth. This process was repeated two additional times to remove residual antibiotic in the culture, before a final re-suspension in 50 μL LB broth. This mixed culture was added in a puddle to an LB agar plate containing no antibiotic and left for ∼20 to 30 min in a laminar flow hood to dry. The puddle was incubated at 27 °C for a minimum of 5 h before an inoculating loop was used to transfer a streak from the center of the puddle to fresh LB agar supplemented with kanamycin and gentamicin. This plate was incubated at 27 °C for 1 to 2 d until distinct colonies could be observed. Cryogenic clonal stocks were produced by growing one colony overnight at 27 °C in the presence of kanamycin and gentamicin and storing the resultant culture in 20% glycerol final volume at −70 °C.

### Scarless Synonymous Augmentation of ntrB Through Two-Step Allelic Exchange

Synonymous variant 289-TAGGCGTC, which is identical to progenitor strain AR2 except for a C:G→G:C mutation at nucleotide 291 on the coding strand of the gene *ntrB*, was created through two-step allelic exchange. This protocol closely followed that outlined by (Hmelo et al. 2015), barring amendments outlined in (Horton et al. 2021), which were made to optimize the protocol for *Pseudomonas fluorescens*. Primers utilized for this work are outlined in supplementary table S1, Supplementary Material online. In brief, an augmented *ntrB* sequence was produced through two PCR steps as outlined above using AR2 as template data, with the amendment that nested primers ntrB_np_F and ntrB_np_R were used to combine the two fragments and add restriction enzyme recognition sequences in the second PCR. The produced insert, and the vector pTS1, were digested with restriction enzymes BamHI-HF and Hind-III-HF (NEB), gel-extracted, and ligated as described above. The ligated plasmid was then transformed into *E. coli* strain ST18 and plated onto LB agar supplemented with 50 μg/mL 5-aminolevulinic acid. The transformed ST18 strain was conjugated with *P. fluorescens* SBW25 derivative AR2 by mixing 750 μL ST18 with 250 μL AR2 and pelleting and puddle mating as outlined above. The conjugative puddle was left to incubate overnight on LB agar supplemented with 50 μg/mL 5-aminolevulinic acid, and a streak was transferred onto LB agar supplemented with tetracycline to select for merodiploid AR2 strains. Two-step recombinants were then selected for by plating on NSLB agar containing +15% w/v sucrose, which selects against merodiploids. Surviving colonies were either *ntrB* C291G mutants or revertants to the AR2 progenitor genotype. Successful mutants were verified by Sanger sequencing of the *ntrB* locus using ntrB_1119_F and ntrB_1119F_R. Cryogenic stocks were then prepared as detailed above.

### Experimental Evolution to Assess Hotspot Potency

We used soft agar motility assays to select for mutations that restore motility, including the T:A→G:C mutations at positions 289 and 683 in *ntrB* (Taylor et al. 2015). To prepare soft agar, we poured 30 mL of molten LB broth (Sigma-Aldrich) containing 0.25% agar (Fisher Scientific) into standard 88 mm diameter petri dishes and left these overnight to solidify. The following morning, the petri dishes were placed in a laminar flow hood with the lids removed for 30 min to dry. A sterile cocktail stick was then used to transfer a single colony to the soft agar. The stick was held perpendicular to the agar and the colony inserted ∼2 mm. Plates were inoculated in the center of the dish aside from strains 289-TCGGGGTC, 289-TAGGCGTC, and 683-TCGGGTC, which were inoculated with four equally separated colonies. The plates were incubated at 27 °C and monitored daily for emergence of motility zones for ≤12 d. The first motile zone to appear per plate was passaged within 24 h of appearance to minimize the impact of clonal interference. This was done by passaging onto fresh agar containing kanamycin and gentamicin by inserting an inoculating loop into the leading edge of the motile zone and streaking to produce single colony forming units. Motile zones observed early during the experiment (≤1 to 2 d) may have evolved from the colony after inoculation on the soft agar, or the mutation may have appeared during the initial growth of the colony. After sampling, the motility plate was discarded to ensure that all emergent motile populations evolved independently.

Passaged plates were incubated for 48 h at 27 °C and a single colony was used as template DNA for a colony PCR with primers ntrB_1119_F and ntrB_1119_R (supplementary table S1, Supplementary Material online). The generated *ntrB* amplicons were purified using a Monarch® PCR DNA clean-up kit (NEB), and Sanger sequencing was performed by Source Bioscience using ntrB_1119_F as a primer. *ntrB* reads provided by Source Bioscience were manually trimmed to remove ∼50 bp from each terminus to remove substitution and 1 bp indel calls of low confidence. Mutations of high confidence were called by aligning the *ntrB* sequence against the SBW25 reference genomic sequence (NCBI Assembly: ASM922v1, GenBank sequence: AM181176.4) using BLAST (https://blast.ncbi.nlm.nih.gov/).

### Analysis of Hotspot Motifs in Salmonella Species

Estimation of relative mutation rates in natural populations of *Salmonella* was based on the data from (Cherry 2023). The number of T→G mutations inferred to have occurred in a sequence context of interest was divided by the number of such sites per genome [a weighted average among many *Salmonella* genomes, as detailed in (Cherry 2023)]. Confidence intervals were calculated assuming a Poisson sampling distribution for the number of mutations.

### Statistical Analysis

Statistical analyses were performed using the R statistical environment (R Core Team 2024), version 4.4.1 (2024-06-14). For experimental data, changes in hotspot potency were determined using Fisher's exact test (fisher.test, from the stats package exact 2 × 2, v4.4.0) with *P* < 0.05 treated as significant. Fold-changes in potency were calculated using Fisher's exact test's odds ratio alongside the produced 95% confidence intervals. Figures were generated using R's *ggplot* package v3.5.1. DNA stability with a G:A mismatch at the mutable T positions was estimated using the R package *rmelting* v1.20.0 (Aravind and Krishna 2024) with the following parameters: nucleic acid concentration = 2 × 10^−6^, Na concentration = 1. G_n_T motif counts and positions were acquired using a custom R script (see Data Availability statement) that screened the SBW25 reference genomic sequence ASM922v1.

## Supplementary Material

msaf183_Supplementary_Data

## Data Availability

Sequencing data and the custom R script generated for this work are available on the Open Science Framework, accessible via DOI: 10.17605/OSF.IO/HSYFX.
