# Supplementary material for: GnT Motifs Can Increase T:A→G:C Mutation Rates Over 1000-fold in Bacteria
Source: Mol Biol Evol. 2025 Aug 4;42(8):msaf183. doi: 10.1093/molbev/msaf183 (PMC12344412; doi:10.1093/molbev/msaf183)
Supplement: msaf183_Supplementary_Data [file msaf183_supplementary_data.pdf]

Supplementary information

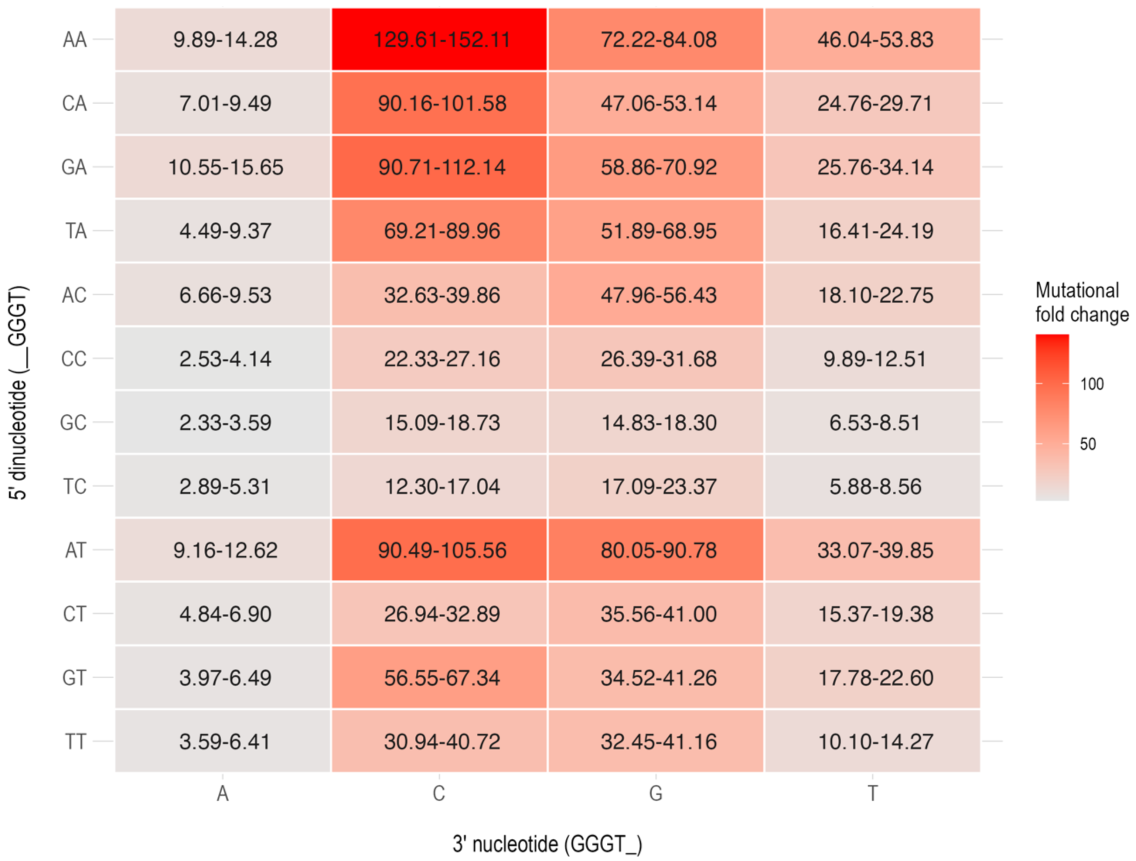

Sup fig 1. The impact of flanking nucleotides neighbouring G_3T tracts on T:A→G:C mutation rates in natural *Salmonella* populations. 95 percent confidence intervals are annotated within each cell.

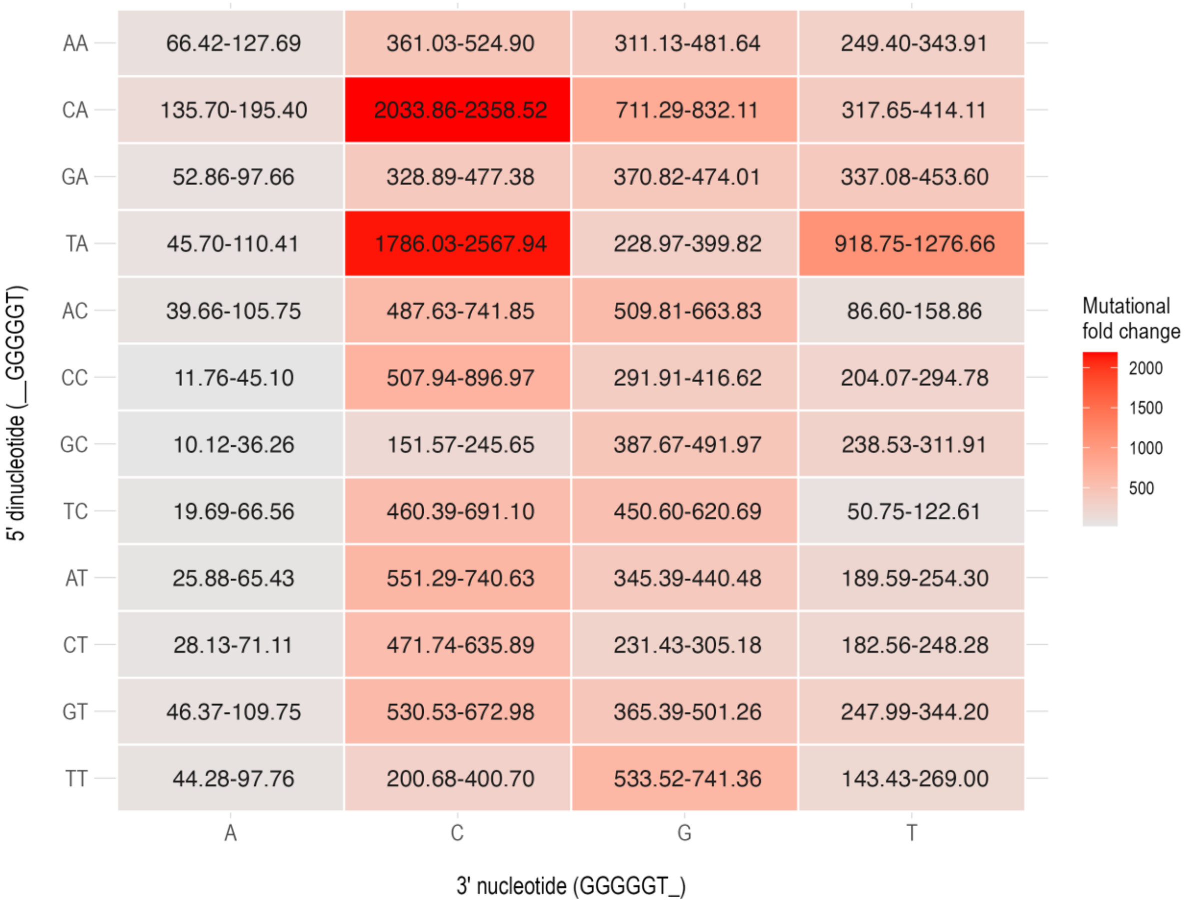

Sup fig 2. The impact of flanking nucleotides neighbouring G_5T tracts on T:A→G:C mutation rates in natural *Salmonella* populations. 95 percent confidence intervals are annotated within each cell.

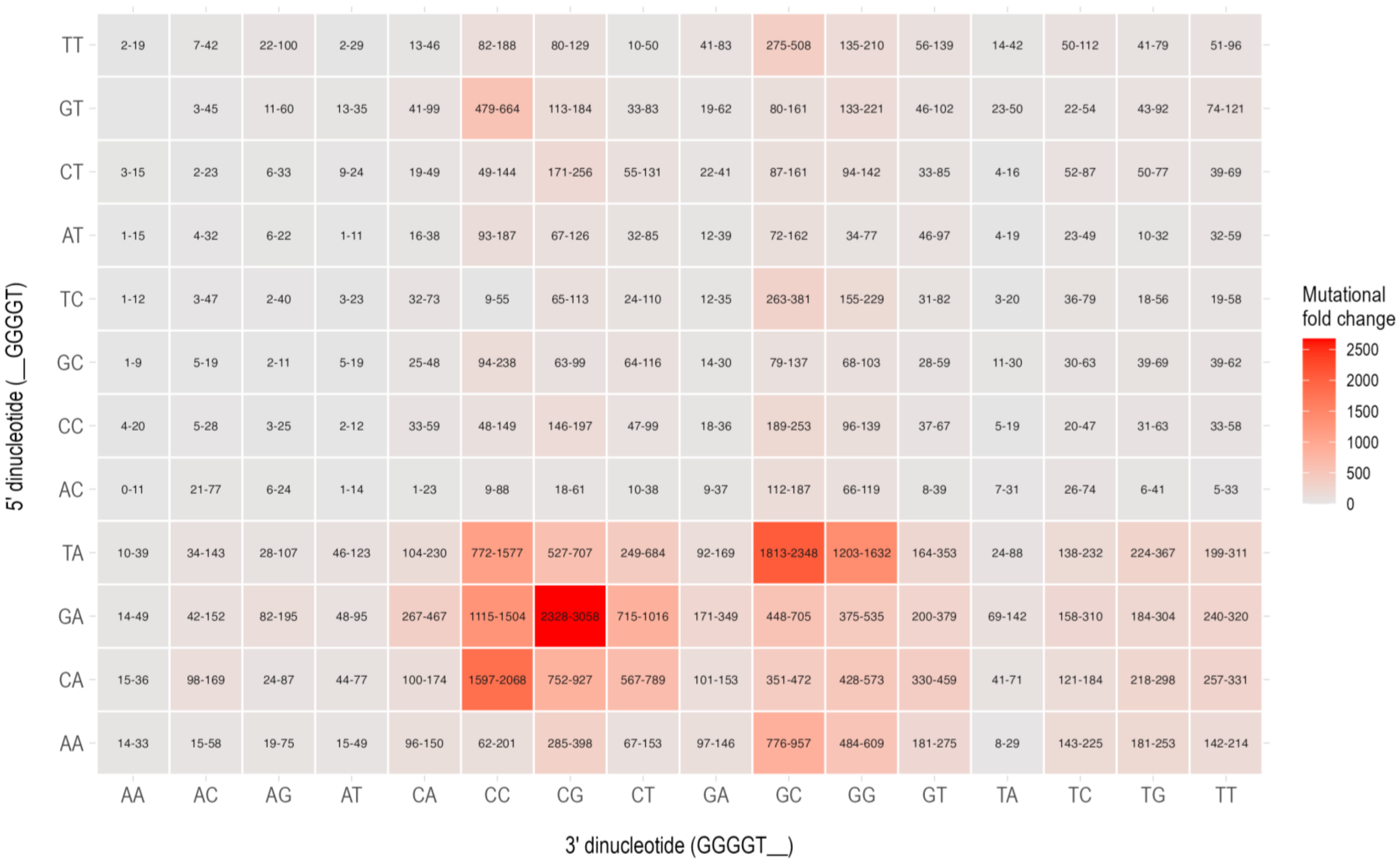

Sup fig 3. The impact of flanking dinucleotides 5’ and 3’ to G_4T tracts on T:A→G:C mutation rates in natural *Salmonella* populations. 95 percent confidence intervals are annotated within each cell, rounded down to the nearest integer. The blank cell reported a mean predicted mutation rate of 0, stemming from 0 observations of this motif.

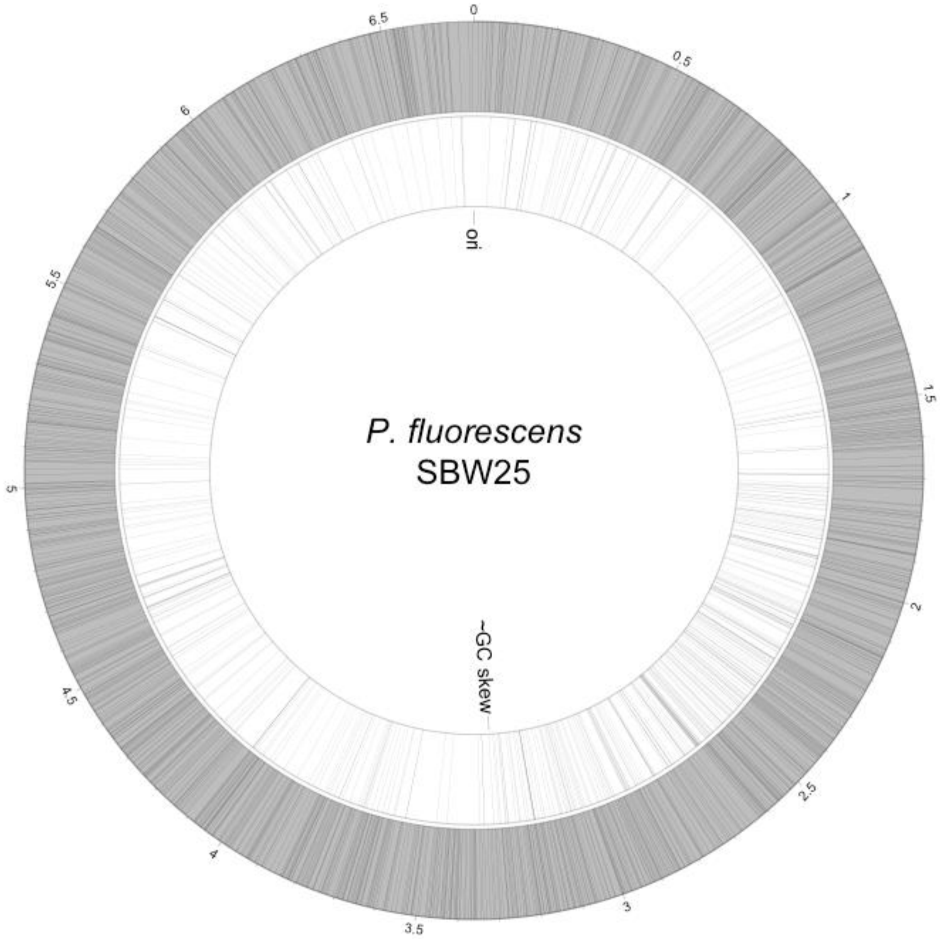

Sup fig 4. Positions of hotspot G_nT motifs within the *P. fluorescens* SBW25 genome. Shown is a circular plot displaying the genomic locations of G_nT motifs with predicted T:A→G:C mutation rates >100-fold higher than the genomic average (using genome assembly ASM922v1). Each motif is highlighted by a black bar 300bp in length (to allow each motif to be visible), with the motif sequence terminating at the end of each window. Motifs found within coding sequence are displayed in the outer ring (grey), and motifs found within intergenic regions are displayed in the inner ring (white). Replichore arms and the respective strand swap were estimated using GC skew as determined by (Silby *et al.*, 2009), approximated at 3.3Mb (~GC skew).

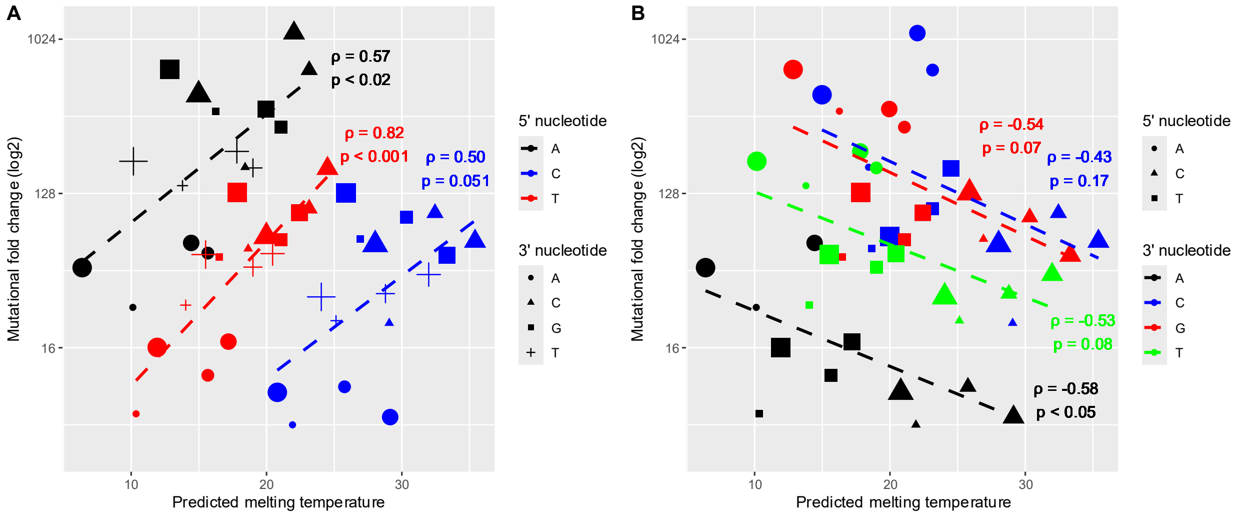

Sup fig 5. Correlation between melting temperature and T:A→G:C mutation rates of G_4T motifs. Mutational fold changes (y-axis; values derived from Fig 3) and predicted melting temperatures assuming a G:A mismatch (x-axis; approximated by nearest-neighbour formulations) were plotted for all G_4T motif variants. rho and p values are plotted next to each determined slope when grouped by the (A) 5' nucleotide, and (B) 3' nucleotide. 5' dinucleotide partner nucleotides are denoted by size in ascending order: A, C, G, T.

Sup table 1. Oligonucleotides used in the study.

**Primer name:**	**Sequence (5'-3'):**	**Primer use:**
ntrBC-HindIII-F	AATTTAAGCTTCACTGTCCGAACAACACTGATC	Outside primers used to generate synonymous ntrBC variant inserts.
ntrBC-SacI-R	AATTTGAGCTCCGGTTCATGGTGCATTGAAGC	
GTT@96-F	CTGACGGTCGACTACGCCGTTACCCCTATCCTGAGCAACG	Inside primer pairs combined with ntrBC-HindIII-F and ntrBC-SacI-R to produce 3' motif variants at ntrB position 289.
GTT@96-R	CGTTGCTCAGGATAGGGGTAACGGCGTAGTCGACCGTCAG	
GTC@96-F	CTGACGGTCGACTACGCCGTCACCCCTATCCTGAGCAACG	
GTC@96-R	CGTTGCTCAGGATAGGGGTGACGGCGTAGTCGACCGTCAG	
CCC@98-F	CTGACGGTCGACTACGCCGTGACCCCCATCCTGAGCAACG	Inside primer pairs combined with ntrBC-HindIII-F and ntrBC-SacI-R to produce 5' motif variants at ntrB position 289.
CCC@98-R	CGTTGCTCAGGATGGGGGTCACGGCGTAGTCGACCGTCAG	
CCG@98-F	CTGACGGTCGACTACGCCGTGACCCCGATCCTGAGCAACG	
CCG@98-R	CGTTGCTCAGGATCGGGGTCACGGCGTAGTCGACCGTCAG	
TCC@230-F	TCACCTTGGTGCGCGACTACGACCCGTCCATTCCCGACGTATTG	Inside primer pairs combined with ntrBC-HindIII-F and ntrBC-SacI-R to produce a 5' motif variant at ntrB position 683.
TCC@230-R	CAATACGTCGGGAATGGACGGGTCGTAGTCGCGCACCAAGGTGA	
CCC@229-F	TCACCTTGGTGCGCGACTACGACCCCAGCATTCCCGACGTATTG	Inside primer pairs combined with ntrBC-HindIII-F and ntrBC-SacI-R to produce a G tract variant at ntrB position 683.
CCC@229-R	CAATACGTCGGGAATGCTGGGGTCGTAGTCGCGCACCAAGGTGA	
CCCTCC@229-230-F	TCACCTTGGTGCGCGACTACGACCCCTCCATTCCCGACGTATTG	Inside primer pairs combined with ntrBC-HindIII-F and ntrBC-SacI-R to produce a G tract and 5' variant at ntrB position 683.
CCCTCC@229-230-R	CAATACGTCGGGAATGGAGGGGTCGTAGTCGCGCACCAAGGTGA	
ntrB_Up_F	GAGAGCTGACCGTTGAAACC	Primer pair used to generate 5' segment of augmented ntrB locus for allelic exchange.
ACG@97-F	CTGACGGTCGACTACGCCGTGACGCCTATCCTGAGCAACG	
ACG@97-R	CGTTGCTCAGGATAGGCGTCACGGCGTAGTCGACCGTCAG	Primer pair used to generate 3' segment of augmented ntrB locus for allelic exchange.
ntrB_Dn_R	ATGGCGCGAAACACTTCCTG	
ntrB_np_F	AATTTGGATCCATGACCATCAGCGATGCACTG	Nested primers used to produce complete augmented ntrB locus for allelic exchange.
ntrB_np_R	AATTTAAGCTTGATCCAGACGGTTTCACTACG	
ntrB_1119_F	GAGGTCCCAATGACCATCAG	Primer pair used for amplicon production of ntrB.
ntrB_1119_R	GACGATCCAGACGGTTTCAC	

References

Silby, M.W. *et al.* (2009) ‘Genomic and genetic analyses of diversity and plant interactions of Pseudomonas fluorescens’, *Genome Biology*, 10(5), p. R51. Available at: https://doi.org/10.1186/gb-2009-10-5-r51.
